# Real-time monitoring with iTLR4 assay identifies ligand-dependent TLR4-TLR4 conformational dynamics

**DOI:** 10.3389/fimmu.2025.1580070

**Published:** 2025-09-01

**Authors:** Sanam Mustafa, Samuel G. Evans, Mark R. Hutchinson

**Affiliations:** ^1^ School of Biomedicine, The University of Adelaide, Adelaide, SA, Australia; ^2^ Australian Research Council Centre of Excellence for Nanoscale BioPhotonics, The University of Adelaide, Adelaide, SA, Australia; ^3^ Institute for Photonics and Advanced Sensing, The University of Adelaide, Adelaide, SA, Australia; ^4^ Davies Livestock Research Centre, The University of Adelaide, Roseworthy, SA, Australia

**Keywords:** dimerisation, real-time, conformational dynamics, bioluminescence resonance energy transfer (BRET), innate immunity, Toll-like receptor 4 (TLR4)

## Abstract

Toll-like receptor 4 (TLR4) plays a pivotal role in the innate immune system by recognizing pathogens and initiating immune responses. Despite extensive research over three decades, current methods lack the resolution to measure ligand-induced TLR4 receptor dynamics at the earliest stages of signaling, relying instead on downstream outputs such as gene expression and cytokine secretion. Here, we present the illuminating TLR4 (iTLR4) assay, a novel Bioluminescence Resonance Energy Transfer (BRET)-based platform that provides real-time insights into TLR4 receptor-level events in live cells. The iTLR4 assay demonstrates, for the first time, that lipopolysaccharide (LPS) induces stable interactions between intracellular domains of TLR4 monomers, with an EC50 of 660 EU/mL. Kinetic analysis revealed a gradual, sustained increase in the BRET signal over time. Additionally, the assay uncovered subtle mechanistic differences among functional antagonists. While all antagonists completely abolished LPS-induced IL-8 secretion, the assay demonstrated that at the receptor level LPS-RS completely inhibited the LPS-induced BRET signal, TAK-242 partially inhibited it and (+)-naloxone potentiated it. The assay also identified potential regulatory roles for CD14 and MD2 in naloxone stereoisomer activity, marking the first report of such mechanistic differences. These findings highlight the unique capabilities of the iTLR4 assay to track nuanced TLR4 receptor dynamics, enabling high-throughput screening of TLR4-specific modulators. This platform provides critical insights into ligand-induced signaling, paving the way for the development of novel therapeutics targeting TLR4-related diseases and advancing our understanding of innate immune responses.

## Introduction

Toll-like receptors (TLRs) are pivotal components of the innate immune system, recognizing pathogen-associated molecular patterns and initiating immune responses (please see recent review) (1). The Nobel Prize awarded to Jules Hoffmann and Bruce Beutler in 2011 underscores the significance of TLRs in the field of medicine. Beyond classical roles in immunity, TLRs, particularly TLR4, are now implicated in diverse physiological and pathological processes, including pain, addiction, metabolism, and reproduction (2–5). These non-classical roles necessitate a deeper understanding of TLR4 receptor dynamics (interactions of temporal and spatial kinetics), signaling and regulation.

Current methods for investigating ligand-induced TLR activation/signaling predominantly rely on downstream measures including changes to transcription factor translocation (e.g. NFkB), gene transcription (e.g. interleukin mRNA), and protein release (e.g. secretion of cytokines like interleukin 8 (IL-8)) (6, 7). These are important and biologically relevant measures of innate immune signaling. However, when measured in the context TLR4 activation, these measurements result in poor sensitivity and specificity as they are often measured minutes or hours after TLR4 activation and do not consider signal amplification or modulation by pathways that are not directly related to TLR activation. As such, they do not provide sufficiently accurate reflection of the events occurring at the first step of TLR activation and cannot be considered to be specific indicators of TLR4 activation. Therefore, tools that provide real-time, receptor-level information are crucial for advancing our molecular understanding and precision therapeutic strategies.

The earliest molecular evidence of TLR activation is the stable dimerization of the extracellular and cytoplasmic domains of TLRs, leading to the recruitment of adapter proteins that facilitate MyD88-dependent and independent signaling pathways (8). TLR4, a key TLR family member, requires co-expression of the accessory proteins CD14 and MD2 for effective ligand-dependent signaling (9, 10). CD14 serves as a co-receptor for several TLRs (11) while MD2 is essential for lipopolysaccharide (LPS) responsiveness forming a multi-receptor complex with TLR4 that recognizes LPS (9).

Traditional methods for assessing TLR4 signaling, such as InvivoGen’s HEK-Blue™-hTLR4 reporter cell line, relies on an endpoint assay measuring NFκB and AP-1 activation via a colorimetric SEAP reporter gene (12). Although informative, endpoint assays do not provide real-time receptor dynamics or kinetic data, instead measuring a signaling event downstream of TLR4 activation. More recent approaches, like the NFκB:eGFP-expressing Jurkat cell line, offer direct GFP measurements but still lack real-time signaling insights of receptor level activation due to delays in transcriptional reporting (13).

Bioluminescence Resonance Energy Transfer (BRET) offers a powerful, sensitive tool for real-time investigation of protein-protein interactions, including receptor signaling and receptor dynamics (14–16). BRET involves a bioluminescent donor that excites a fluorescent acceptor when in close proximity (within 10 nm), producing an emission that signifies protein interaction. This real-time detection capability allows for precise quantification of ligand-dependent specific protein-protein interaction and decoupling events (receptor dynamics) without confounding factors like photobleaching (please see (15, 17, 18) for examples).

BRET has previously been utilized for the study of interactions between TLR2 and signaling adaptor proteins MyD88 and TIRAP (19). However, due to constitutive proximity between proteins of interest resulting in a measurable BRET signal in the absence of the TLR2 ligand, Pam3CSk4, this application of BRET was not pursued further. It is also worth mentioning that fluorescence resonance energy transfer (FRET), an approach that utilizes a similar concept to BRET for the study of protein-protein interactions, has been used to investigate the TLR4 TIR dimerization interface (20), TLR4 trafficking to lipid rafts (21) and the interaction between TLR4 and TLR2 (22). FRET, however, to the best of our knowledge, has not been utilized to investigate the real-time ligand-dependent activation of TLR4-TLR4 dimerization and C-terminal conformational changes.

In this study, we introduce the illuminating TLR4 (iTLR4) assay, a novel real-time assay for monitoring early-stage dimerization and C-terminal conformational changes of TLR4, providing information about receptor dynamics and signaling. We generated genetically tagged TLR4 constructs by introducing either Nanoluciferase (bioluminescent donor) or Venus (fluorescent acceptor) to the C-terminal region of TLR4. By utilizing these BRET-compatible TLR4 constructs, we achieved real-time measurement of TLR4 signaling initiation in living cells upon LPS exposure. This assay provides the first real-time biological evidence of TLR4 receptor dynamics and mechanistic insights of antagonist function, offering significant advancement for detailed investigation and drug screening in TLR4-related pathologies.

## Materials and methods

### Materials

Human Embryonic Kidney 293-FT (HEK293) cells were purchased from Life Technologies (VIC, AUS). Dulbecco’s modified Eagle’s medium (DMEM) - high glucose (D5671) and Dulbecco’s Phosphate Buffered Saline (PBS) (D8537) were purchased from Merck (AUS). Phenol red free DMEM – high glucose, HEPES (21063029), Trypsin (Gibco) (15400054), Nunc™ MicroWell™ 96-well, Nunclon delta-treated, flat bottom microplates (136101), and Costar^®^ black, clear bottom 96-well assay plates (165303), Lipofectamine 3000 (L3000001), Fetal Bovine Serum (FBS) (Cat# 16000044), 4’,6-Diamdino-2-Phenylindole, Dihydrochloride (DAPI) (D1306) and Alexa Fluor 680 conjugated Wheat Germ Agglutinin (WGA) (W32465) were purchased from ThermoFisher (AUS). LPS from Escherichia coli (E.coli) 0111:B4 purified by phenol extraction, LPS from Rhodobacter sphaeroides (LPS-RS) and CLI-095 (TAK-242) were purchased from InvivoGen (CA, USA). LPS, LPS-RS and TAK-242 were prepared as per manufacturer’s instructions and stored at -20°C. LPS was made to 1x10^^6^ EU/mL in PBS before preparation of final working concentration. Nano-Glo^®^ Luciferase assay substrate (N1130) was purchased from Promega (Aus). Cell Signalling Technology^®^ anti-mouse NFkB p65 (6956S), anti-rabbit NFkB p65 (8242S) and anti-rabbit HA-Tag (C29F4), were purchased from New England BioLabs (AUS). Donkey anti-rabbit 594 (A21207), donkey anti-mouse 594 (A21203) and donkey anti-rabbit 488 (A21206) were purchased from Life Technologies (AUS). Fluoro-Gel, water-based mounting medium was purchased from ProSciTech (QLD, AUS). Human IL-8 ELISA kit (Cat# 555244) was purchased from BD-Bioscience (USA). (+)- and (-)-Naloxone, kindly donated by Dr. Kenner Rice (Chemical Biological Research Branch, National Institute on Drug Abuse and National Institute on Alcohol Abuse and Alcoholism, National Institutes of Health, Rockville, MD) were prepared in dH_2_O and stored at 4°C for 48 hrs. DAMGO (Tocris bioscience) was purchased from *In Vitro* Technologies (AUS) and reconstituted in 100% DMSO to 1mM and stored at –20°C.

### Constructs

Generation of HA-TLR4-Venus (TLR4-Venus) and HA-TLR4-NLuc (TLR4-NLuc) constructs: Human TLR4A (TLR4) cDNA, purchased from Invivogen, was subcloned into pcDNA3 plasmids containing coding regions for Venus or NanoLuciferase (NLuc) (kindly gifted by Prof Kevin Pfleger, University of Western Australia, Australia) via HindII/NotI or HindIII/XhoI to generate Venus or NLuc C-terminal tagged TLR4 respectively. An HA (Influenza Hemaglutinin; Tyr, Pro, Tyr, Asp, Val, Pro, Asp, Tyr, Ala) epitope was also introduced on the N-terminal by PCR.

Generation of FLAG-MOP-Nluc (MOP-Nluc) construct: FLAG-MOP (kindly gifted by Prof. Brigitte Kieffer, University of Strasbourg, France) was subcloned into the same pcDNA3 plasmids containing coding regions for NanoLuciferase (NLuc) used above via HindIII/XhoI to generate NLuc C-terminal tagged FLAG-MOP.

All cloning was performed by the GSEx facility at the University of Adelaide. MD2 (pEFIRES) and CD14 (pcDNA3) constructs were kindly gifted by Prof. Clare Bryant (University of Cambridge, UK). β-arrestin 2-Venus (pcDNA3) and GRK2 (pcDNA3) were kindly gifted by Prof Kevin Pfleger, University of Western Australia, Australia.

### Cell culture

HEK293 cells were maintained in culture media (DMEM supplemented with 4.5 g/L glucose, 10% (w/v) FBS, 2 mM L-Glutamine, 50 U/ml penicillin and 50 mg/mL streptomycin) at 37°C in a 5% CO_2_ humidified incubator. Cultures were passaged every two to three days using 0.05% trypsin to maintain sub 80% confluency.

### Transfections

Transfections were conducted 24 hours (h) following seeding of 350,000 cells per well (6 well plate) in 2 ml of culture media without penicillin and streptomycin. Transfections were performed when cells reached 60-70% confluency.

#### TLR4

Lipofectamine™ 3000 transfection reagent was used as per the manufacturer’s protocol with the following deviations. Lipofectamine™ 3000 was used at a ratio of 3:4 (0.75 µL/1 µg DNA), and P3000 reagent at a ratio of 1:1 (1 µL/1 µg DNA). The transfection mix was incubated for 25 minutes (min) before drop-wise addition to cells. 1.5 mL of media was replaced 5.5 h following the addition of DNA, and cells re-plated 24 h later.

Immunocytochemistry and ELISA construct validation – 1 µg/well of DNA consisting of 1 µg of either TLR4-Venus or TLR4-NLuc or 0.5 µg each of TLR4-Venus and TLR4-NLuc. For experiments involving LPS treatment, a total of 1.6 µg/well was added, which included 300 ng of CD14 and MD2 along with TLR4 combinations mentioned above.

BRET assays - 1.6 µg/well of DNA consisting of 50 ng TLR4-Venus, 50 ng TLR4-NLuc, 275 ng of CD14 and MD2 and 950 ng pcDNA3.1 plasmid. When a construct was omitted, the equivalent amount of pcDNA3 was added to ensure equal addition of total cDNA. Transfections omitting TLR4-Venus constructs were prepared as a negative control for BRET assays.

#### MOP

jetPEI^®^ was used as per the manufacturers protocol. 500 ng of DNA was added per well consisting of 16.7 ng of Flag-MOPR-Nluc, 317.3 ng β-arrestin-2-Venus and 166 ng GRK. The jetPEI: DNA ratio was 2:1. The transfection mix was incubated for 30 min before drop-wise addition to cells. Cells were re-plated 24 h later.

### BRET assay

Transfected cells were replated in triplicates at 100,000 cells per well in white (Nunclon Delta-Treated) and black clear-bottom 96 well Poly-D-Lysine coated plates. Cells were plated in phenol red free DMEM (4.5 g/L glucose, (5%) (w/v) FBS, 2 mM L-glutamine, 25 mM HEPES) and left overnight to adhere. The next day, Venus fluorescence from cells plated in the black microplate was measured on a BMG CLARIOstar plate reader (BMG Labtech, Ortenberg, Germany) to assess transfection success (excitation 497–15 nm, emission 540–20 nm).

Agonist treatments - Supernatant on transfected cells (white plate) was replaced with 80 µl of Nano-Glo^®^ Luciferase Assay Substrate prepared in phenol red free DMEM at 1:1000. The plate was incubated for 5 min at 37°C before luminescence baseline readings at 475–30 nm and 535–30 nm were recorded for approximately 12 min (3 readings recorded every 247 s) on a BMG CLARIOstar plate reader. LPS, Pam3CSK (TLR2 agonist) (+)-, (-)-Naloxone and DAMGO were diluted from stocks in phenol red free DMEM to 5X concentration, 20 µl of the appropriate compound was added to each well and the plate was measured for 2 h total. A vehicle only control was included on all plates.

Antagonist treatment - Supernatant from transfected cells (white plate) was replaced with 100 µl of antagonist or vehicle for 2 h at 37 °C. Supernatant was then removed and replaced with either antagonist (prepared in phenol red free DMEM), vehicle or agonist (LPS 1000 EU/mL or DAMGO 10 mM) plus antagonist for 2 h. Supernatant was then removed and replaced with 100 µl of Nano-Glo^®^ Luciferase Assay Substrate prepared in phenol red free DMEM at 1:1000. The plate was incubated for 5 min at 37°C and luminescence was measured at 475–30 nm (donor) and 535–30 nm (acceptor) for approximately 20 min (5 readings recorded every 247 s) on a BMG CLARIOstar plate reader.

BRET signal data processing - The 535–30 nm acceptor signal was divided by the 475–30 nm donor signal and multiplied by 1000 to calculate a BRET signal for each well and replicates averaged. For real time experiments the BRET signals were then normalized to baseline before proceeding. Vehicle treated BRET signal was subtracted from treatment BRET signal to obtain the ligand-induced BRET signal (for pre-treatment antagonist experiments, 5 measurements were recorded, and BRET signals were averaged to obtain one value per plate).

### Immunocytochemistry

Transfected cells were replated into 24 well plates containing Poly-D-Lysine coated 13 mm coverslips (pre-sterilized in 70% ethanol) at 250,000 cells/well and left to adhere overnight. Cells were incubated with vehicle or LPS (1000 or 2000 EU/mL) for 60 min, including a 10 min incubation with WGA-680 (1:400) prior to fixation.

Cells were fixed by the addition of ice cold 4% paraformaldehyde containing 5% sucrose for 15 min immediately following removal of media. Cells were washed (x3) in 1X PBS. Following washes, cells were permeabilized with 0.2% Triton-X 100 in 1X PBS for 10 min, followed by a 10 min block in 3% skim milk and 0.1% Tween 20 in 1X PBS. Primary antibodies were added in blocking solution O/N at 4°C (rabbit anti-HA, 1:500; anti-mouse NFkB, 1:500; anti-rabbit NFkB, 1:1000). Cells were washed (x3) in 1X PBS before addition of secondary antibodies and DAPI for 60 min at room temperature in block solution (donkey anti-rabbit 594, 1:500; donkey anti-mouse 594, 1:500; donkey anti-rabbit 488, 1:500; DAPI, 1:10,000). Cells were washed (x3) in 1X PBS and placed on microscope slides with Fluoro-Gel and allowed to dry. Cells were imaged on a ZEISS LSM980 - Airyscan Confocal and associated ZEN blue viewer software (Ver3.7).

### IL-8 ELISA

Transfected cells were replated into 96 well plates (100,000 cells/well) and left to adhere overnight. Supernatant was aspirated and cells treated with LPS in culture media (100 µL) or vehicle (media only) for 4 h (for antagonist experiments, cells were pre-treated for 2 h, followed by 4 h antagonist/LPS co-treatment). 80 mL of supernatant was removed at the conclusion of treatments, and IL-8 content was measured using a Human IL-8 ELISA kit according to the manufacturer’s instructions.

### Statistics

All statistical tests were performed in GraphPad Prism software (version 10.4.0).

Unpaired t-tests were used to compare AUC of baseline-corrected BRET signals between ligand (LPS, PAM3SK) and vehicle treatments as well AUC of ligand-induced BRET signal between transfections (following LPS) and when comparing DAMGO to co-treatments with (+)- or (-)-naloxone. T- and P-values were reported for all t-tests as (t(degrees of freedom) = [t-value], p = [p-value]), with relevant variables being reported as (M = mean; SD = standard deviation). AUC was calculated to include area above Y = 0 (BRET signal of 0).

A mixed-effects model with Geisser-Greenhouse correction and Šidak’s multiple comparisons test was used to anaylze the effects of time and treatment on LPS and vehicle-treated baseline-corrected BRET signals. Both F and P-values from mixed-effects analysis are reported as (F(degrees of freedom (interaction effect), degrees of freedom (within groups) = [F-value], p = [p-value]).

A one sample t-test was used to compare the LPS-induced BRET signal at each time point to the corresponding baseline (y = 0). One sample t-tests were also used to compare the % response of each antagonist concentration to vehicle treated response (y = 100).

An ordinary one-way ANOVA with Dunnett’s comparison test was used to compare AUC of naloxone-induced BRET signal between transfections. AUC was calculated to include area above Y = 0 (BRET signal of 0). An ordinary one-way ANOVA with Tukey’s comparison test was used to compare treatment groups for all ELISA data. Both F and P-values of the one-way ANOVA are reported as (F(degrees of freedom (between groups), degrees of freedom (within groups) = [F-value], p = [p-value]).

A non-linear fit (four parameter variable slope) was used to determine EC50 in concentration-response experiments. A non-linear fit (three parameter) was used to determine IC50 in concentration-response experiments.

## Results

### Functional validation of BRET-compatible TLR4 constructs: construct expression and NFκB activation and IL-8 secretion

**Figure 1:**
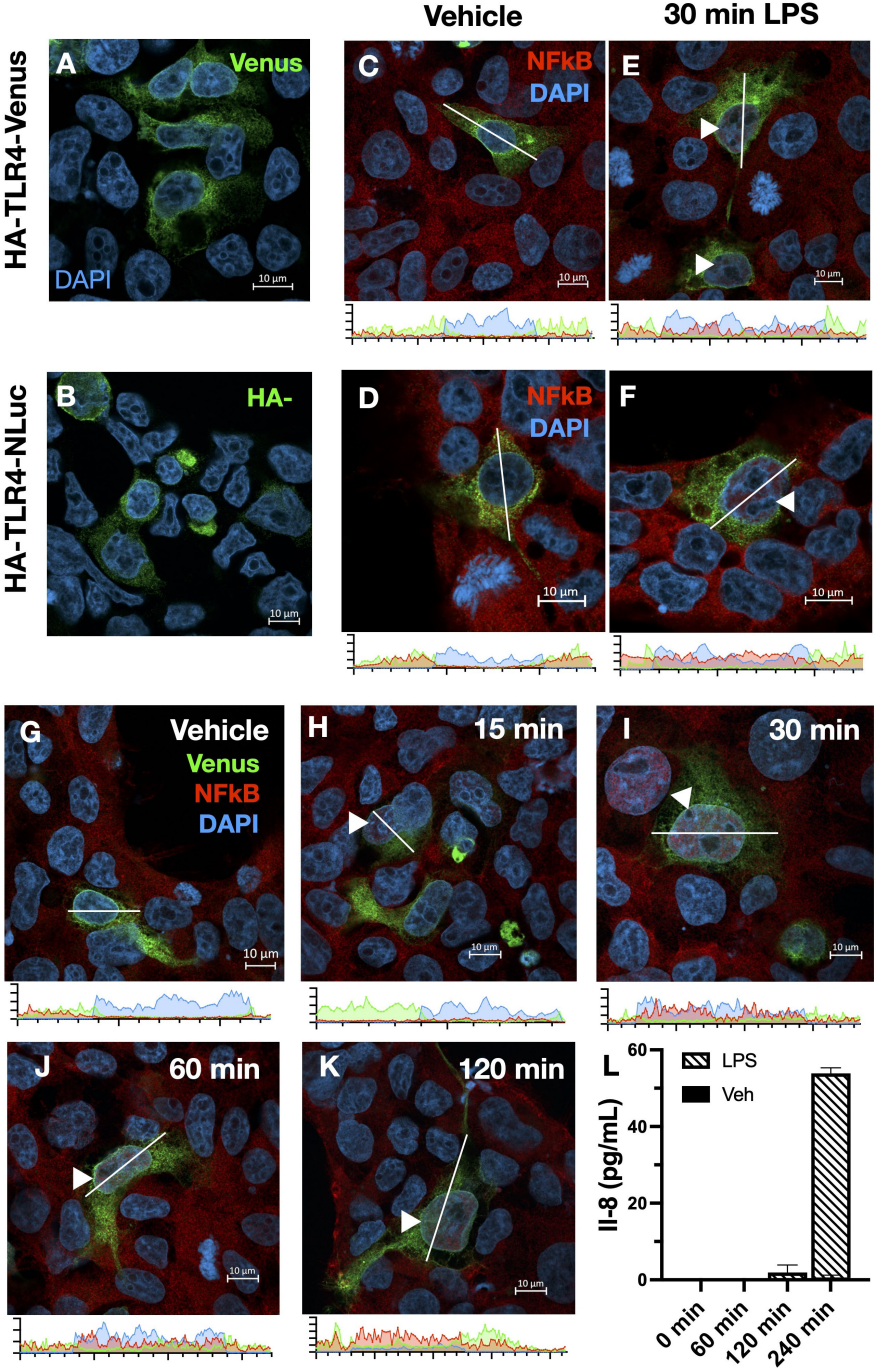
TLR4-NLuc or TLR4-Venus constructs can be expressed in HEK293 cells and are functional. Validation of TLR4-NLuc or TLR4-Venus constructs transiently transfected in HEK293 cells with signaling accessory proteins MD2 and CD14 **(A-F)**. TLR4-Venus expression **(A)**, TLR4-NLuc **(B)** expression confirmed by Venus visualization and HA staining, respectively. Cells co-expressing CD14 and MD2 with either TLR4-Venus **(C, E)** or TLR4-Nluc **(D, F)** were exposed to LPS (1000 EU/mL) or vehicle for 30 min and stained for NFkB. NFkB translocation to the nucleus was observed following a 30 min LPS treatment but not in vehicle-treated cells. TLR4-Venus and TLR4-NLuc co-transfected in HEK293 cells with signaling accessory proteins MD2 and CD14 **(G-L)**. LPS-induced (2000 EU/mL) NFkB nuclear translocation was observed at 15-, 30-, 60- and 120-min post-administration **(G-K)**. An ordinary one-way ANOVA with Tukey’s comparison test revealed that LPS-induced IL-8 secretion was detectable following 240 min (p < 0.0001) of LPS treatment (2000 EU/mL) **(L)**. Intensity data displayed under images **(C-K)** represent fluorescence intensity along the white line. Arrows depict nuclei with visible NFkB translocation. Images are representative of 3 separate transfections. n = 3 for ELISA data, error bars represent SEM.

To validate the functionality of the BRET-compatible TLR4 constructs, HEK293FT (HEK293) cells were transiently transfected with either the BRET donor HA-TLR4-NLuc (TLR4-NLuc) or BRET acceptor HA-TLR4-Venus (TLR4-Venus), along with necessary signaling accessory proteins MD2 and CD14. Expression of TLR4 was confirmed by Venus fluorescence and HA staining (**Figures 1A, B**). Following LPS treatment (1000 EU/mL, 4 h), IL-8 secretion was detected in the supernatant (data not shown). Additionally, the ability of tagged TLR4 to activate NFκB was assessed by observing NFκB translocation in cells treated with LPS (1000 EU/mL) for 30 min (**Figures 1C-F**). To confirm co-expression of TLR4-Nluc and TLR4-Venus with MD2 and CD14 did not impact TLR4 function, transfected HEK293 cells were treated with LPS (2000 EU/mL) and NFκB translocation to the nucleus was observed in a time-dependent manner (**Figures 1G-K**). NFκB translocation was observed from 15 min following LPS treatment (**Figure 1H**). Additionally, IL-8 secretion was detected in the supernatant of these cells and a one-way ANOVA confirmed that there was an effect of LPS compared to vehicle (F(6,14) = 1.04, p < 0.0001) (**Figure 1L**). Dunnett’s multiple comparison analysis revealed a significant difference from baseline at 240 min (p < 0.0001, 95% C.I. = -57.68, -50.12) but at no earlier time points.

Together, this data confirms that the genetically engineered tags do not impact TLR4 expression or function. Importantly, cells expressing both forms of tagged TLR4 (BRET donor and acceptor) retain the ability to activate NFκB and secrete IL-8 in response to LPS.

### LPS-induced changes in BRET ratios: real-time monitoring of BRET signal

**Figure 2:**
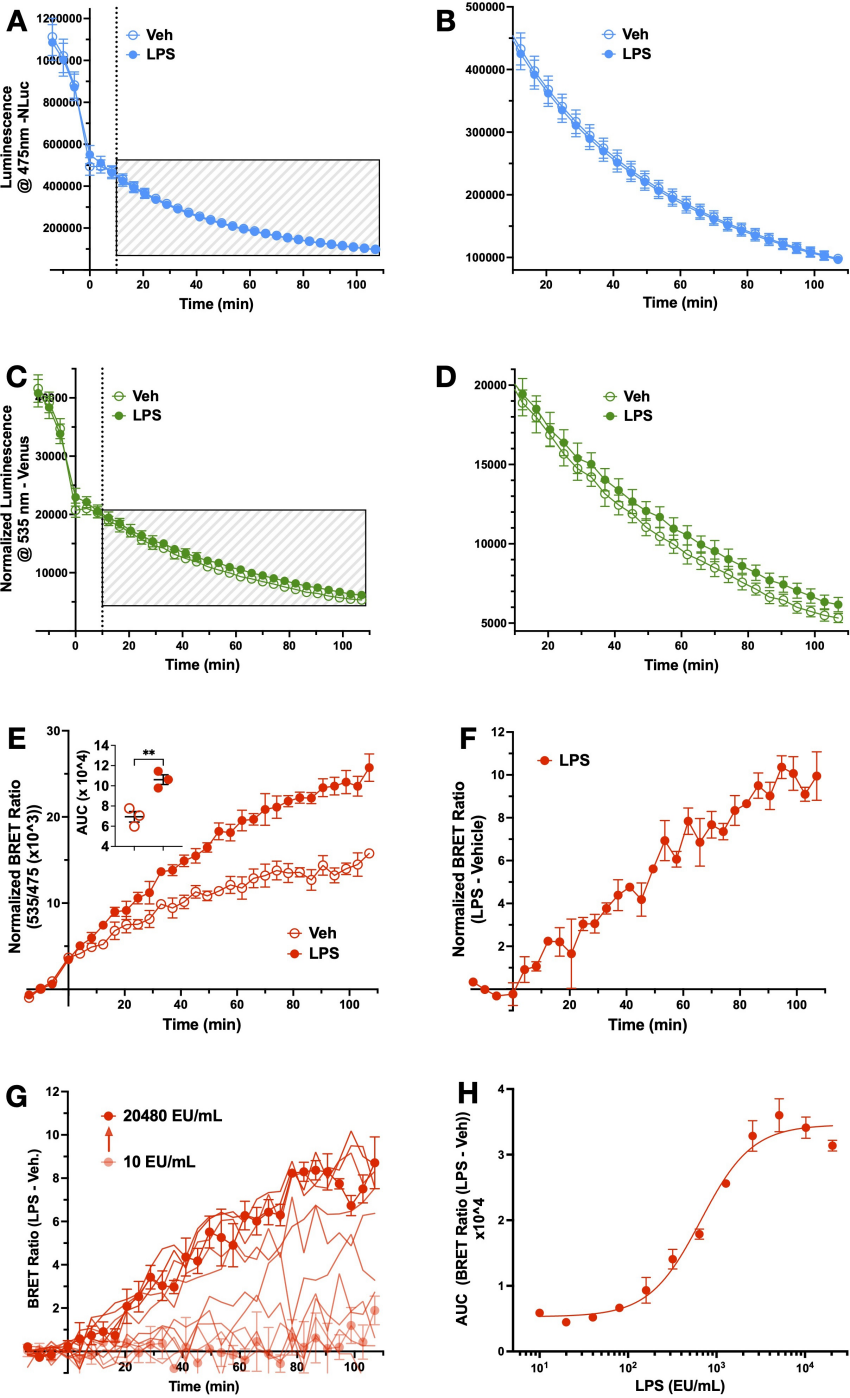
LPS alters BRET ratios in HEK293 cells co-transfected with TLR4-Venus, TLR4-NLuc, MD2 and CD14. NLuc **(A)** and Venus **(C)** emission was measured over a 2 h period following incubation with LPS (2000 EU/mL) or vehicle in HEK293 cells transiently co-expressing TLR4-NLuc, TLR4 Venus, MD2 and CD14. **(B, D)** represent boxed areas of **(A, C)**, respectively. **(E)** represents the ratio of NLuc to Venus signal (x1000). An unpaired t-test revealed a significant difference between AUC of LPS and vehicle-treated groups (p = 0.007) (**E**, inset). A ligand-induced BRET signal was observed by subtracting vehicle from LPS-treated groups **(F)**. The LPS-induced BRET signal was concentration-dependent **(G)**. The AUC of each concentration was used to establish an EC50 (660 EU/mL) **(H)**. n = 3 separate transfections for all data points, error bars represent SEM.

HEK293 cells transfected with TLR4-Venus, TLR4-NLuc, MD2, and CD14 were incubated with either LPS (2000 EU/mL) or vehicle over a two-hour period. Throughout the treatment, luminescence emitted by Nluc (**Figures 2A, B**) and fluorescence emitted by Venus (**Figures 2C, D**) tags was measured. After calculating the ratio of the two signals (BRET signal), the data was normalized to baseline and an unpaired t-test was used to compare AUC. A significant effect (t(4) = 5.2, p = 0.007) of LPS (M = 10.61, SD = 0.83) compared to vehicle (M = 6.93, SD = 0.90) (**Figure 2E**) was observed. A mixed effects model was used to compare the effect of time and treatment (LPS or vehicle) on the BRET signal. This revealed an effect of time (F(2.9, 11.4) = 264.5, p < 0.0001) and a time x treatment interaction (F(29,116) = 20.6, p < 0.0001), but no treatment effect (F(1,4) = 6.3, p = 0.07). *Post-hoc* analysis revealed no difference at any time point between vehicle and LPS-induced BRET signal. Subtraction of the vehicle-treated signal from the LPS-treated signal revealed a steady, but slow increase in the LPS-induced BRET signal over time (**Figure 2F**). A one sample t-test confirmed a significantly higher signal post-addition of LPS (t(4) = 24.3, p = 0.002) at 13 min (M = 2.30, SD = 0.16) than baseline (y = 0), and then continuously from 25 min until the end of the recording (107 min) (**Figure 2F**). The BRET signal was confirmed to be LPS concentration-dependent, with an EC_50_ of 660 EU/mL (**Figures 2G, H**).

This data confirmed that by utilizing BRET to measure the proximity of the NLuc and Venus tags on TLR4-NLuc and TLR4-Venus proteins, an LPS-dependent change in the proximity of the C-terminal regions, of at least two differentially tagged, TLR4 receptors could be measured in real-time. Therefore, this BRET signal represents the first LPS-induced step in TLR4 signaling and this data provides the foundation for the illuminating TLR4 (iTLR4) assay, a novel platform that enables real-time measurement of LPS-induced TLR4 signaling using BRET.

### LPS concentration-dependent BRET signal: BRET signal and accessory protein dependence

**Figure 3:**
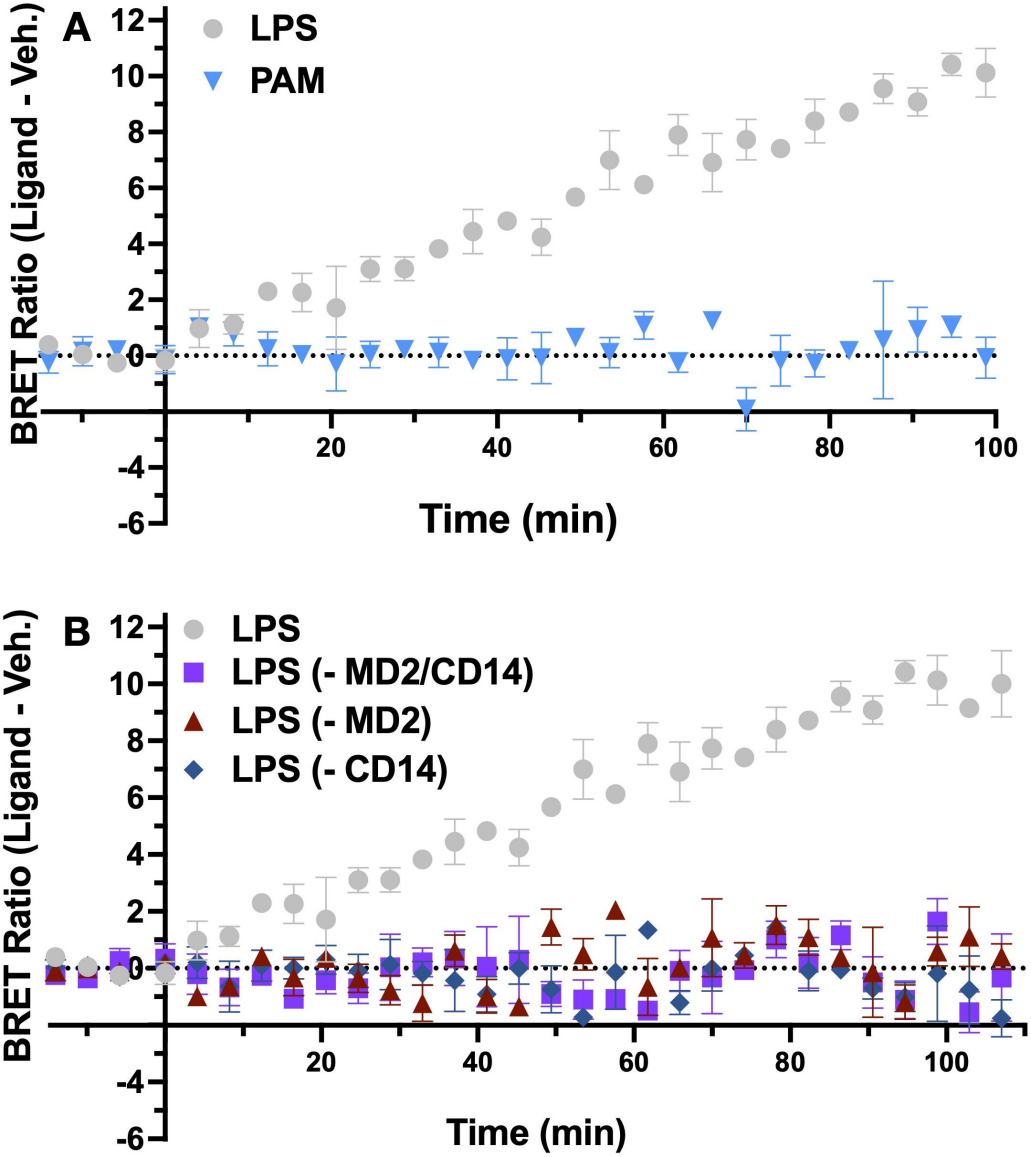
LPS-induced TLR4-NLuc-TLR4-Venus BRET signal is MD2 and CD14 dependent. HEK293 cells transiently transfected with TLR4-Venus, TLR4-Nluc, MD2 and CD14 did not produce a BRET signal when exposed to TLR2 agonist Pam3CSK4 (100 ng/mL) (p = 0.87) **(A)**. No change in BRET signal was observed following LPS (2000 EU/mL) treatment when accessory proteins MD2 (p = 0.93), CD14 (p = 0.99) or both (p = 0.98) were omitted from the transfection **(B)**. All p-values represent results of unpaired t-tests between AUC of vehicle and ligand treated cells. n = 3 for all data points, error bars represent SEM.

To confirm the LPS-induced BRET signal observed in the iTLR4 assay (**Figure 2**) was the result of a specific TLR4 ligand-induced interaction of TLR4-NLuc and TLR4-Venus, we utilized Pam3CSK4, a TLR2 agonist not reported to have activity at TLR4 (23, 24). An unpaired t-test was performed to compare the AUC of baseline corrected BRET signal generated by Pam3CSK4 and vehicle treated cells. There was no significant difference between treatment with Pam3CSK4 (100 ng/mL) (M = 6.91, SD = 0.66) and vehicle (M = 6.81, SD = 0.77); t(4) = 0.18, p = 0.87 (**Figure 3A**).

As mentioned earlier, MD2 is essential for recognizing LPS. To further confirm the BRET signal detected in the iTLR4 assay was due to a specific interaction of LPS, HEK293 cells were transfected with TLR4-Venus, TLR4-NLuc and CD14 in the absence of MD2. An unpaired t-test was performed to compare the AUC of baseline corrected BRET signal generated by LPS and vehicle treated cells. When MD2 was removed, no difference was observed (t(4) = 0.09, p = 0.93) between the LPS (M = 6.58, SD = 0.58) and vehicle (M = 6.61, SD = 0.34). A similar observation was made when MD2 was re-introduced but CD14 was absent (LPS (M = 7.91, SD = 3.91), vehicle (M = 7.88, SD = 3.77); t(4) = 0.02, p = 0.99) and also when both CD14 and MD2 were excluded (LPS (M = 7.24, SD = 3.79), vehicle (M = 7.31, SD = 3.76); t(4) = 0.02, p = 0.98) (**Figure 3B**).

To summarize, the iTLR4 assay is capable of detecting a BRET signal that is specifically induced by a TLR4, not TLR2, agonist. By utilizing iTLR4 assay, the critical dependence of the LPS-induced BRET signal, and hence receptor dynamics, on the co-expression of CD14 and MD2 was confirmed.

### Inhibition of LPS-induced BRET signal by LPS-RS and TAK-242 but not by (+)-naloxone: differential effects of TLR4 inhibitors

**Figure 4:**
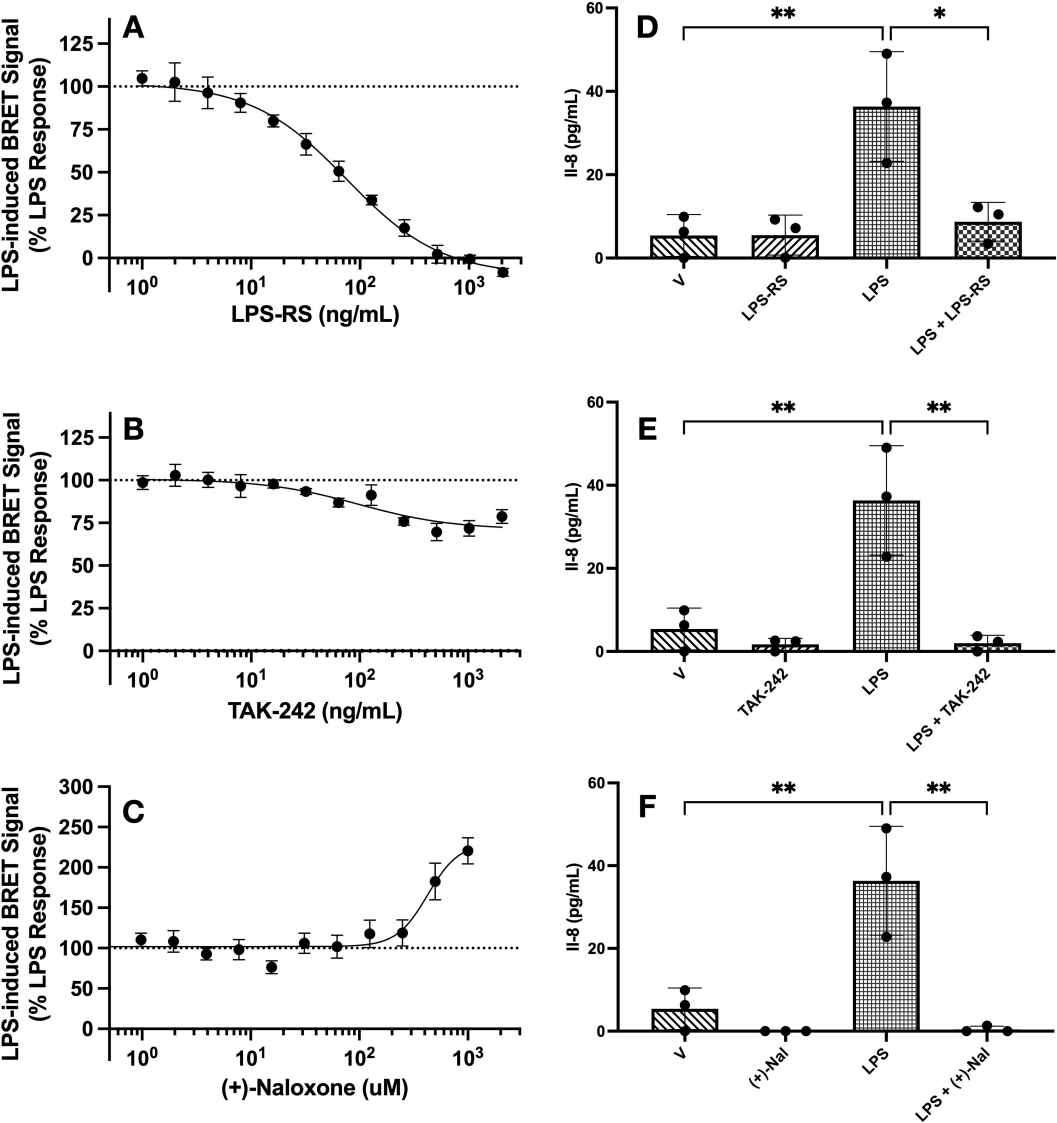
LPS-induced TLR4-NLuc-TLR4-Venus BRET signal and IL-8 release is significantly inhibited by LPS-RS and TAK-242 but (+)-naloxone potentiates the BRET signal whilst inhibiting IL-8 release. TLR4-Venus, TLR4-NLuc, MD2 and CD14 were transiently transfected in HEK293 cells. Cells were pre-treated with increasing concentrations of either LPS-RS **(A)**, TAK-242 **(B)** or (+)-naloxone **(C)** for 2 h followed by the addition of LPS (1000 EU/mL) for a further 2 h. Following incubation, Nano-Glo^®^ was added and 5 measurements taken to determine BRET ratios. To assess if significant changes in the LPS-induced BRET signal were observed in the presence of LPS-RS, TAK-242, or (+)-naloxone, a one sample t-test was performed. LPS-RS completely attenuated the LPS-induced BRET signal **(A)**. TAK-242 caused partial attenuation **(B)** and (+)-naloxone potentiated the LPS-induced BRET signal **(C)**. In parallel, transfected cells were pre-treated with LPS-RS (1 µg/mL) **(D)**, TAK-242 (1 µg/mL) **(E)** or (+)-naloxone (5 mM) **(F)** for 2 h before a 4 h treatment with LPS (1000 EU/mL). An ordinary one-way ANOVA with Tukey’s comparison test confirmed all TLR4 antagonists attenuated LPS-induced IL-8 responses in transfected cells. * = p < 0.05, ** = p < 0.01. n = 3 for all data points except B (n = 4) and C (n = 5), error bars represent SEM.

To understand the impact of TLR4 antagonists on LPS-induced BRET signal, HEK293 cells expressing TLR4-Venus, TLR4-NLuc, MD2, and CD14 were pre-treated with TLR4 antagonists LPS-RS, TAK-242 and (+)-naloxone before exposing them to LPS (1000 EU/mL) in the iTLR4 assay. For each concentration of antagonist tested, percentage of response compared to vehicle treated cells (no antagonist exposure) was calculated. A one sample t-test was performed to compare the response percentage of each antagonist concentration with response of cells not exposed to an antagonist (response percentage = 100%). Attenuation of LPS-induced BRET signal by LPR-RS was concentration dependent (IC50–75 ng/mL) and complete by 500 ng/mL. An effect was first observed at 16 ng/mL ((M = 79.91, SD = 5.98); (t(2), = 5.82, p = 0.03)). (**Figure 4A**). In contrast, TAK-242 only partially attenuated the LPS-induced BRET signal. The highest inhibition (of 30%) was observed at 500 ng/mL. The lowest concentration to produce significant inhibition was 32 ng/mL ((M = 93.35, SD = 3.433); (t(3), = 3.87, p = 0.03)). With the exception of 128 ng/mL, all concentrations tested above 32 ng/mL also resulted in significant attenuation of the LPS-induced BRET signal (**Figure 4B**). (+)-naloxone significantly potentiated the LPS-induced BRET signal at 500 (M = 182.70, SD = 51.01) and 1000 (M = 220.50, SD = 36.53) µM (t(4), = 3.62, p = 0.02 and t(4), = 7.38, p = 0.002, respectively) (**Figure 4C**). Maximally effective concentrations of these antagonists were then utilized to investigate their impact on LPS-induced IL-8 secretion from HEK293 cells expressing TLR4-Venus, TLR4-NLuc, MD2, and CD14. All three antagonists significantly attenuated LPS-induced IL-8 release. One-way ANOVA revealed a significant effect of treatment for LPS-RS (1 µg/mL) (F(3,8) = 0.10, p = 0.003), TAK-242 (1 µg/mL) (F(3,8) = 2.18, p = 0.0009) and (+)-naloxone (5 mM) (F(5, 12) = 2.02, p < 0.001) (**Figure 4D-F**). LPS-RS was a more potent inhibitor of IL-8 secretion at 200 ng/mL compared to TAK-242 (data not shown).

Although all antagonists were capable of inhibiting LPS-induced IL-8 secretion, the iTLR4 assay enabled the identification of key mechanistic differences in the action of (+)-naloxone from LPS-RS and TAK-242. In a concentration-dependent manner, (+)-naloxone increased the LPS-induced BRET signal between TLR4-NLuc and TLR4-Venus. This is in contrast to the actions of LPS-RS and TAK-242, which inhibit the LPS-induced BRET signal.

### iTLR4 assay reveals naloxone-induced TLR4 C-terminal conformational changes: high naloxone concentrations induce augmented BRET signals in the absence of accessory proteins

**Figure 5:**
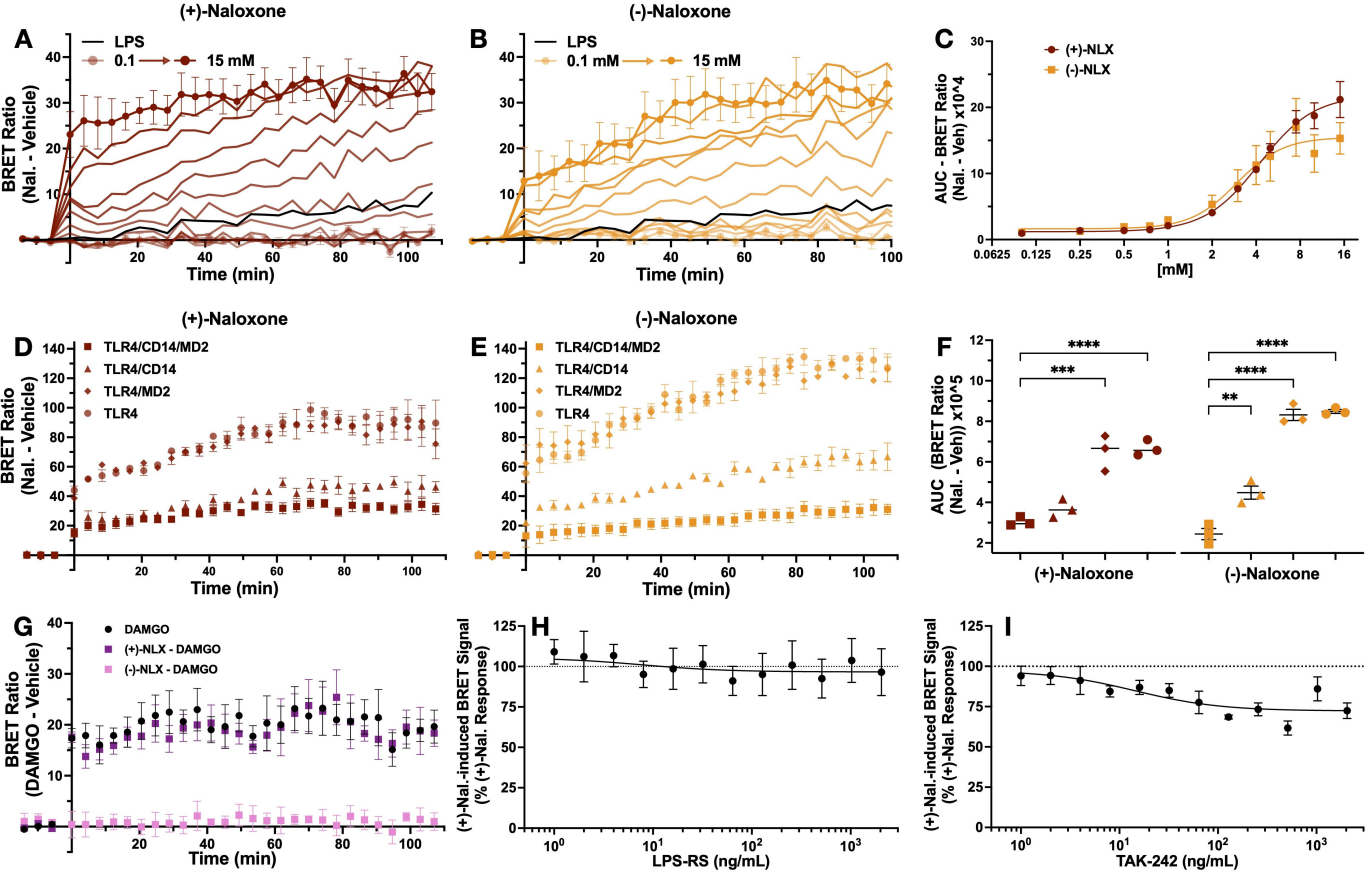
Naloxone-induced TLR4-NLuc-TLR4-Venus BRET signal is neither dependent on MD2 and CD14 or completely inhibited by LPS-RS and TAK-242. TLR4-Venus, TLR4-NLuc, MD2 and CD14 were transiently transfected in HEK293 cells. These cells were exposed to increasing concentrations of (+)- **(A)** and (-)-naloxone **(B)**. The rate of BRET signal increase was observed to be concentration-dependent with an EC50 of 4.2 and 3 mM, respectively **(C)**. Compared to cells that express both MD2 and CD14, an ordinary one-way ANOVA with Dunnett’s comparison revealed the AUC of the naloxone (10 mM)-induced BRET signal was significantly increased when CD14 was absent for both stereoisomers (p < 0.0001) **(D, E)** and to a lesser extent when MD2 was absent in response to (-)-naloxone (p = 0.001) **(E)** but not (+)-naloxone (p = 0.05) **(D)**. **(F)** represents the AUC of naloxone-induced BRET signal for each transfection treated with (+)- and (-)-naloxone. A mu opioid receptor - β-arrestin2 BRET system was used to confirm the activity of each isomer. An unpaired t-test revealed 100 mM of (-)-naloxone (p = 0.005) but not (+)-naloxone (p = 0.79) attenuated DAMGO (10 mM)-induced BRET signal **(G)**. To assess if significant changes in the (+)-naloxone-induced BRET signal were observed in the presence of LPS-RS or TAK-242, a one sample t-test was performed. At no concentration tested did LPS-RS inhibit (+)-naloxone-induced BRET signal **(H)**. In contrast, TAK-242 resulted in partial inhibition starting at 16 ng/mL (p = 0.02). n = 3 separate transfections for all data points (except **H, I**, n = 4), error bars represent SEM.

To further investigate the actions of naloxone on the TLR4 BRET signal, the effects of both (+)- and (-)-naloxone stereoisomers were examined in the iTLR4 assay. For both (+)- and (-)-naloxone, the AUC of ligand-induced BRET signal was concentration-dependent (**Figures 5A, B**), with an EC_50_ of 4.2 and 3.0 µM, respectively (**Figure 5C**). To determine the role of accessory proteins in the action of (+)- and (-)-naloxone, MD2 and CD14 were removed from the transfection, as described above. A one-way ANOVA revealed a significant effect of transfection for both (+) and (-)-naloxone on AUC of ligand-induced BRET signal ((F(3,8) = 35.7, p < 0.0001) and (F(3,8) = 132.5, p < 0.0001)). Dunnett’s multiple comparisons analysis revealed that for (+)-naloxone-induced BRET signal, a significant increase in AUC was observed by removal of CD14 (p = 0.0001, C.I. = -4.73, -2.17) and both CD14/MD2 (p < 0.0001, C.I. = -4.90, -2.35) compared to cells expressing both CD14 and MD2. However, the removal of only MD2 had no effect (p = 0.38, C.I. = -1.93, 0.63) (**Figures 5D, F**). In contrast, the AUC of (-)-naloxone-induced BRET signal was significantly increased by removal of CD14 (p < 0.0001, C.I. = -6.93, -4.83), MD2 (p = 0.001, C.I. -3.10, -0.10) and both CD14/MD2 (p < 0.0001, C.I. = 7.10, 5.00) when compared to cells co-transfected with both MD2 and CD14 (**Figure 5E, F**). It should be noted that the magnitude of increase was smaller in the group without MD2 (M = 4.48) compared to groups without CD14 (M = 8.32) and both accessory proteins (M = 8.49). Dunnett’s multiple comparisons revealed that compared to the group without MD2, a significant increase in AUC of (-)-naloxone-induced BRET signal was observed in both the group without CD14 (p < 0.0001, C.I. = -4.89, -2.78) and without both CD14/MD2 (p < 0.0001, C.I. = -5.01, -2.95).

To confirm the pharmacological actions of both isomers were not compromised, 100 µM (+)- and (-)-naloxone were tested for their ability to antagonize a DAMGO-induced BRET signal between mu opioid receptor-Nanoluciferase (MOP-NLuc) and β-arrestin2-Venus. Confirming literature that opioid receptors are stereoselective for (-)-isomers (25, 26), only (-)-naloxone was able to attenuate DAMGO-induced BRET signal between MOP-NLuc and β-arrestin2-Venus. Unpaired t-tests revealed a significant difference between the AUC of DAMGO-induced BRET signal (M = 13.20, SD = 3.44) compared with signal resulting from cells treated with DAMGO and (-)-naloxone (M = 1.51, SD = 0.99)(t(4) = 5.66, p = 0.005). In contrast, no effect was observed when compared to cells treated with DAMGO and (+)-naloxone (M = 12.44, SD = 3.02)(t(4) = 0.29, p = 0.79) (**Figure 5G**).

Next TLR4 antagonists LPS-RS and TAK-242 were utilized to investigate their ability to inhibit the (+)-naloxone induced BRET signal. Antagonist effects were analyzed as described above. LPS-RS had no effect on (+)-naloxone induced BRET signal (**Figure 5H**). In contrast, TAK-242 partially attenuated this signal, akin to its inhibitory effect on the LPS-mediated BRET signal (**Figure 4**). One sample t-test revealed inhibition at 8 ng/mL (M = 84.43, SD = 8.73) compared to (+)-naloxone only control (y = 100%) (t(3), = 4.51, p = 0.02). With the exception of 16 and 1024 ng/mL, all higher concentrations tested also resulted in significant attenuation of the (+)-naloxone-induced BRET signal (**Figure 5I**).

In a concentration-dependent manner, both stereoisomers of naloxone induce a BRET signal between TLR4-NLuc and TLR4-Venus, which is augmented in the absence of CD14 and in the case of (-)-naloxone, also in the absence of MD2. To our knowledge, the data generated through the iTLR4 assay is the first-time subtle differences in the role of accessory proteins, CD14 and MD2, in the actions of (+)- and (-)-naloxone stereoisomers at TLR4 has been reported.

## Discussion

In this study we introduced the iTLR4 assay, a creative application of Bioluminescence Resonance Energy Transfer (BRET), to measure real-time ligand-induced TLR4 signaling. The LPS-induced BRET signal, measured by the iTLR4 assay, represents activation of the first step in TLR4 signaling, where the C-terminal regions of TLR4 receptors undergo a conformational change enabling close proximity (within 10 nm) indicative of protein-protein interactions. Additionally, the iTLR4 assay has enabled insight into key molecular differences in functional TLR4 antagonists LPS-RS, TAK-242 and (+)-naloxone, including subtle differences in naloxone stereoisomer activity at TLR4, which have not been identified before. By tracking these interactions dynamically, we can gain insights into the early signaling events and receptor dynamics of TLR4 that were previously inaccessible with traditional endpoint assays.

### Implications for TLR4 research and beyond

TLR4 is involved in a broad spectrum of biological processes beyond innate immunity, including pain, addiction, reproduction, and cancer (2, 5, 27–29). These non-classical roles of TLR4 extend its significance across various physiological and pathological contexts, necessitating precise tools to dissect its signaling mechanisms. Despite its extensive study, tools to investigate TLR4 signaling at the earliest activation points have been lacking. Traditional methods focus on downstream events such as cytokine secretion or transcription factor translocation, which, while informative, are not specific to TLR4 and can be influenced by various signaling pathways and cellular events. This lack of specificity hinders directly correlating ligand binding with receptor activation/deactivation and subsequent signaling events.

The iTLR4 assay overcomes these limitations by providing real-time receptor-level insights. Our study confirmed the functionality of BRET-compatible TLR4 constructs in HEK293 cells, which initiated NFκB translocation and IL-8 secretion upon LPS treatment (**Figure 1**). The LPS-induced BRET signal, observed only when MD2 and CD14 were present, indicates the necessity of these accessory proteins for LPS-dependent TLR4 signaling (**Figures 2**, **3**). This assay thus provides a direct measure of ligand action on TLR4-TLR4 C-terminal conformation dynamics and therefore receptor signaling, bypassing the confounding effects of downstream signaling pathways.

### Insights into TLR4 receptor dynamics

Previous studies have suggested that TLR4 forms transient dimers prior to ligand activation, with subsequent ligand-induced conformational changes facilitating receptor activation (30). More recent studies indicate TLR4 exists as a mix of monomers and dimers, with LPS promoting dimer formation and stabilization (31, 32). Latty and colleagues provide evidence that agonist stimulation, such as LPS, leads to the formation of TLR4 dimers and stabilization of preformed dimers which then allows signaling pathway activation (31). A similar conclusion was made by Kruger and colleagues where they estimated, in the absence of LPS stimulation, around 50% of TLR4 was in a monomeric state and the remaining forming dimers (32). Our study did not differentiate if the LPS-dependent BRET signal observed is due to dimerization of monomeric tagged TLR4 proteins, the stabilization/C-terminal conformational change of preformed TLR4 dimers into an active state, or a combination of both. Nevertheless, our results revealed that TLR4 signaling by LPS is a dynamic process, likely involving both the formation of new dimers and the stabilization of pre-existing ones. The iTLR4 assay sensitivity to LPS concentration underscores the stabilization of TLR4 in its activated conformation, a crucial step in the initiation and propagation of TLR4 signaling pathways. These findings support the use of the iTLR4 assay as a tool for real-time monitoring of TLR4 receptor dynamics.

Notably, the TLR4-NLuc-TLR4-Venus BRET signal increased gradually over time, contrasting with the rapid response seen in other receptor-receptor BRET studies such as the histamine H3 receptor homodimerisation, which reaches maximal BRET signal by 10 min (33). This suggests a more regulated, almost cautious molecular response to LPS, potentially allowing for differential TLR4-mediated inflammation depending on the nature and duration of the stimulus. This gradual activation contradicts the traditional “all-or-nothing” view of TLR4 signaling, proposing instead that the kinetics of TLR4 receptor dynamics are ligand-dependent (34). The ability to monitor these kinetics in real-time opens new avenues for understanding how different ligands and conditions influence TLR4 signaling. Indeed, iTLR4 assay opens up the possibility to further investigate the ligand-dependent kinetics of TLR4 signaling in future studies.

The iTLR4 assay demonstrated high specificity for TLR4 agonists, with the BRET signal observed only following treatment with a TLR4 agonist (LPS) and not with a TLR2 agonist (Pam3CSK4) (**Figure 3**). The absence of a BRET signal in the presence of Pam3CSK4 confirms that the observed interactions are specific to TLR4 and not due to random protein interactions or artifacts of the overexpression system. This specificity is crucial for accurately studying TLR4 dynamics and evaluating potential TLR4-targeted therapeutics.

Our study also highlighted the differential inhibitory effects of LPS-RS and TAK-242 on TLR4 signaling. LPS-RS completely attenuated both the LPS-mediated BRET signal and IL-8 secretion in a concentration-dependent manner, whereas TAK-242 only partially inhibited the BRET signal by around 30% at the higher concentrations tested (500 and 1000 ng/mL), but fully attenuated IL-8 secretion (**Figure 4**). Unlike LPS-RS, a competitive antagonist, binding in the LPS binding site (35), TAK-242 (a selective TLR4 inhibitor also known as resatorvid) has been reported to act on the Cys747 in the TLR4 intracellular helix C of the TIR domain (36–38). Although Takashima and colleagues have previously reported that TAK-242 does not impact LPS-induced TLR4-TLR4 conformational change and dimerization, their study utilized both a protein fragment complementation assay and an antibody approach published in 2003 (37, 39). Antibody detection, although a valuable approach, is not as sensitive as the BRET based approach adopted in this study. Taken together with the knowledge Cys747 is located in the helix C, which forms a part of the intracellular dimer interface (40), it is therefore not surprising that the highly sensitive iTLR4 assay was able to identify a change in TLR4-Nluc-TLR4-Venus BRET signal (indicative of TIR domain conformational change/dimerization) following TAK-242 treatment. TAK-242’s ability to inhibit intercellular signaling, such as IL-8 secretion observed in our study, is well established (36, 41). Surprisingly, (+)-naloxone did not inhibit the BRET signal between TLR4-NLuc and TLR4-Venus, but high concentrations of this functional antagonist unexpectedly increased the BRET signal (**Figure 4**). Interestingly, the (+)-naloxone induced BRET signal was amplified in the absence of CD14 but not in the absence of MD2 (**Figure 5**). This suggests MD2 does not play a role in (+)-naloxone mediated actions, but CD14 may have a potential regulatory role. However, in the case of (-)-naloxone, although the absence of CD14 resulted in a similar increase in BRET signal to (+)-naloxone, the absence of MD2 also augmented the BRET signal (albeit, not to the same extent). To our knowledge, this is the first time differences in naloxone stereoisomers have been identified with respect to TLR4 activity. As LPS-RS was not able to inhibit the (+)-naloxone-induced BRET signal (**Figure 5**), this data suggests that (+)-naloxone is binding a site that is unique from the LPS binding site. Interestingly, TAK-242 exhibited a modest inhibition of the (+)-naloxone-induced BRET signal as it did for LPS, suggesting that helix C dimerization was weakened in its presence and TAK-242’s site of activity is accessible during both LPS and (+)-naloxone interaction with TLR4. The impacts of LPS and TLR4 antagonists on TLR4-Nluc-TLR4-Venus have been summarized in **Table 1**.

**Table 1:** Schematic illustrating proposed dynamic TLR4 conformational states.

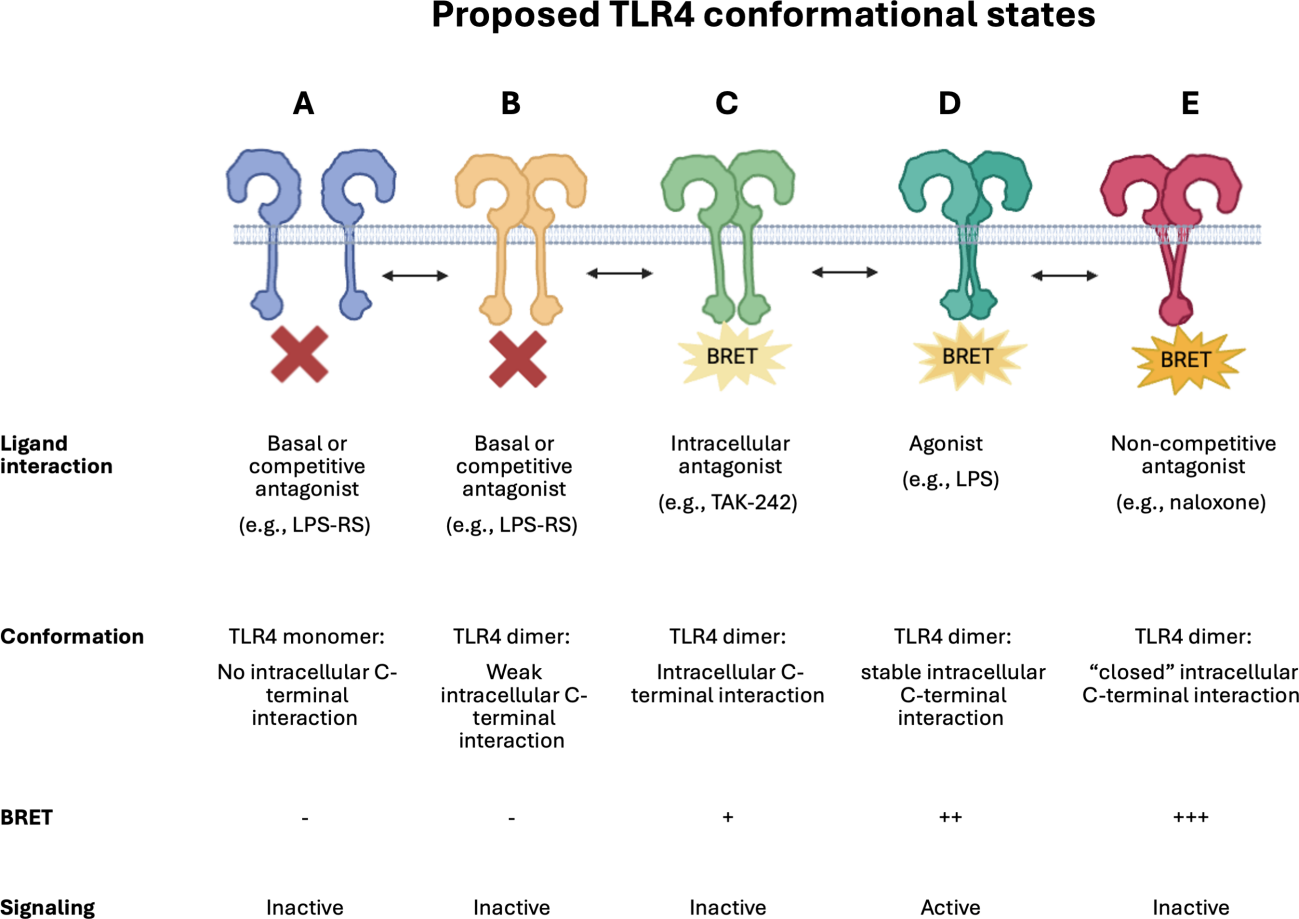

A simplistic representation of the proposed dynamic conformational changes identified by this study. TLR4 monomers (**A**) and dimers (**B**) that do not form close C-terminal interactions will not be detected by the iTLR4 assay and are reported to be inactive with respect to downstream signaling. LPS results in TLR4 C-terminal domains, harboring the donor and acceptor BRET tags, to undergo a conformational change (**D**), bringing the tags in close proximity and resulting in a detectable BRET signal in the iTLR4 assay. This BRET signal can be completely blocked by competitive antagonists such as LPS-RS (**A** or **B**) or partially by intracellular acting antagonists such as TAK-242 (**C**). It is proposed that naloxone results in a strong BRET signal due to inducing a conformational change in bringing the C-terminal regions of TLR4 in much closer proximity (**E**). This very close proximity results in the C-terminal regions, critical for downstream signaling, to be hidden due to the ‘closed’ dimer state and therefore, preventing signaling. Illustration created in BioRender.com.

### Future directions and applications

The findings reported in our study underline the importance of appreciating TLR4 signaling as more nuanced than the simplistic view often represented in textbooks: monomers equating to inactivity and dimers equating to the presence of an active complex. It is more likely that numerous conformational states exist within the TLR4 dimer state, which are stabilized in a ligand dependent manner, to achieve inactive or varying degrees and type of active states. It is important to consider also the microenvironment in which these receptor conformational states occur, as it is not unreasonable to predict, amongst other factors, that the pH, temperature or indeed the co-expression of other proteins may also impact TLR4’s ability to form active or inactive dimer conformations to then modulate intracellular signaling. Although pH and temperature were not investigated in this study, the iTLR4 assay identified that the presence of MD2 and CD14 hampered naloxone’s ability to induce a BRET signal.

It is postulated that (+)-naloxone acts as a functional antagonist by locking the TLR4-intracellular dimer interface in a rigid “closed” or “off” conformation preventing TLR4’s intracellular domains from interacting with key intracellular signaling proteins to activate signaling pathways. The partial inhibition of both the LPS and (+)-naloxone induced BRET signal by TAK-242 suggests that while TAK-242 effectively inhibits downstream signaling events like cytokine secretion, it does not fully prevent the receptor C-terminal conformational change detected by the iTLR4 assay. This highlights the complexity of TLR4 signaling modulation and the need for assays that can identify subtle differences in mechanisms of action at the level of the receptor complex. Although not investigated in this study, future studies which include eritoran, an extensively studied synthetic lipodisaccharide acting as a competitive antagonist in a similar manner to LPS-RS, may identify subtle differences in its mechanism of action, with respect to receptor C-tail conformation (42). This may provide additional insights and understanding of the conformational states detected by the iTLR4 assay. Please see **Table 1** which incorporates a schematic summarizing proposed dynamic TLR4 conformational states. Needless to say, this is highly unlikely to be an exhaustive list of the numerous ligand-dependent conformation changes that occur in nature.

The iTLR4 assay presents a significant advancement for the real-time monitoring of TLR4 receptor dynamics and signaling. Future studies could explore the impact of additional accessory proteins, such as the LPS binding protein (LBP), on TLR4 receptor dynamics. LBP, for instance, can enhance the sensitivity of TLR4 to LPS by presenting it more effectively to the TLR4-MD2-CD14 complex, potentially amplifying the BRET signal (43). Investigating such interactions could provide deeper insights into the modulation of TLR4 signaling by accessory molecules and co-receptors.

Although iTLR4 presents an exciting opportunity to identify TLR4-specific activators, when assessing antagonists, it will be important not to dismiss antagonists that do not exhibit full inhibition or amplify the TLR4-NLuc-TLR4-Venus BRET signal as they may have a similar mechanism of action to TAK-242 or naloxone. It is important to also note that the BRET signal reported in this study does not represent nor measure all TLR4 C-terminal interaction events. A signal is only obtained when differentially tagged TLR4 (TLR4-NLuc and TLR4-Venus) dimerize or undergo a conformational change. The iTLR4 assay, by its nature, is not able to measure a signal when TLR4-NLuc-TLR4-NLuc or TLR4-Venus-TLR-Venus dimerization or conformational change occurs. Therefore, when evaluating the data obtained from these studies, it is important to consider that the measured signal is likely to equate to approximately a third of total TLR4 C-terminal interaction events. As HEK293 cells lack endogenous TLR4, tagged TLR4-endogenous TLR4 dimerization does not need to be considered. A further consideration would be the relative transfection efficiency of each cDNA construct. Additionally, one limitation of utilizing a BRET based assay is the compatibility of colored or fluorescent compounds. Colored compounds can interfere with the BRET signal by decreasing or increasing the signal, leading to false positive or negative signals. Fluorescent compounds can also falsely change the BRET signal if they share similar spectral properties as the acceptor or donor. The inclusion of controls or the avoidance of colored or fluorescent compounds can overcome these issues. As a ‘first generation’ TLR4 receptor signaling assay there are opportunities for future enhancements that, for example, build upon sensitivity or utilize cell lines stably expressing protein constructs to allow more rapid assessment of TLR4 signaling.

As such, the assay’s potential for high-throughput screening of TLR4-specific activators and inhibitors makes it a valuable tool for drug discovery and the development of novel therapeutics targeting TLR4 signaling pathways. High-throughput screening using the iTLR4 assay could identify new compounds that modulate TLR4 activity, offering therapeutic potential for conditions where TLR4 is implicated, such as sepsis, chronic inflammatory diseases, and certain cancers. The ability to screen large libraries of compounds in a real-time, receptor-specific manner accelerates the identification and optimization of promising drug candidates.

The iTLR4 assay provides a sensitive, precise, and real-time approach for investigating TLR4 signaling. Its application in various physiological and pathological contexts holds promise for advancing our understanding of TLR4 roles and developing targeted interventions for diseases involving TLR4 signaling. By providing a tool to study the early events in TLR4 signaling with high specificity and sensitivity, the iTLR4 assay stands to significantly impact both basic research and clinical applications, facilitating the development of next-generation therapeutics.

## Data Availability

The raw data supporting the conclusions of this article will be made available by the authors, without undue reservation.
